# Chronic glucocorticoid exposure causes brown adipose tissue whitening, alters whole‐body glucose metabolism and increases tissue uncoupling protein‐1

**DOI:** 10.14814/phy2.15292

**Published:** 2022-05-04

**Authors:** Jocelyn S. Bel, T. C. Tai, Neelam Khaper, Simon J. Lees

**Affiliations:** ^1^ Biotechnology Program Lakehead University Thunder Bay Ontario Canada; ^2^ 7890 Northern Ontario School of Medicine Thunder Bay Ontario Canada; ^3^ 7728 Biology Laurentian University Sudbury Ontario Canada; ^4^ 7728 Chemistry and Biochemistry Laurentian University Sudbury Ontario Canada; ^5^ 7728 Biomolecular Sciences Program Laurentian University Sudbury Ontario Canada; ^6^ Biology Lakehead University Thunder Bay Ontario Canada

**Keywords:** corticosterone, insulin resistance, metabolic syndrome, mirabegron, obesity, UCP‐1

## Abstract

Adipose tissue (AT) has been found to exist in two predominant forms, white and brown. White adipose tissue (WAT) is the body's conventional storage organ, and brown adipose tissue (BAT) is responsible for non‐shivering thermogenesis which allows mammals to produce heat and regulate body temperature. Studies examining BAT and its role in whole‐body metabolism have found that active BAT utilizes glucose and circulating fatty acids and is associated with improved metabolic outcomes. While the beiging of WAT is a growing area of interest, the possibility of the BAT depot to “whiten” and store more triglycerides also has metabolic and health implications. Currently, there are limited studies that examine the effects of chronic stress and its ability to induce a white‐like phenotype in the BAT depot. This research examined how chronic exposure to the murine stress hormone, corticosterone, for 4 weeks can affect the whitening process of BAT in C57BL/6 male mice. Separate treatments with mirabegron, a known β3‐adrenergic receptor agonist, were used to directly compare the effects of corticosterone with a beiging phenotype. Corticosterone‐treated mice had significantly higher body weight (*p* ≤ 0.05) and BAT mass (*p* ≤ 0.05), increased adipocyte area (*p* ≤ 0.05), were insulin resistant (*p* ≤ 0.05), and significantly elevated expressions of uncoupling protein 1 (UCP‐1) in BAT (*p* ≤ 0.05) while mitochondrial content remained unchanged. This whitened phenotype has not been previously associated with increased uncoupling proteins under chronic stress and may represent a compensatory mechanism being initiated under these conditions. These findings have implications for the study of BAT in response to chronic glucocorticoid exposure potentially leading to BAT dysfunction and negative impacts on whole‐body glucose metabolism.

## Graphical abstract

Comparing the mechanism of chronic corticosterone on adipose tissue cell signaling with mirabegron, a known β3‐adrenergic receptor (β3AR) agonist. The process in which mirabegron increases UCP‐1 is well known, however, it has not yet been determined how chronic corticosterone Also results in increased protein expression of UCP‐1.

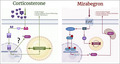

## 1 INTRODUCTION

Adipose tissue (AT) has evolved from originally being thought of as just an energy storage organ to being a complex component of the endocrine system. Adipose tissue’s main function is to store excess energy as triglycerides within the lipid droplets of its cells (adipocytes) in order for them to be accessible to the organism when energy demands are not met (Glantschnig et al., 2019). The conventional storage organ, white adipose tissue (WAT), makes up 95% of AT mass (Kahn et al., 2019), and is composed of unilocular adipocytes that hold triglycerides within their large lipid droplet (Glantschnig et al., 2019; Smith & Horwitz, 1969). White adipose tissue is most commonly linked with obesity and type 2 diabetes mellitus (T2DM) due to an imbalance between energy intake and energy expenditure (Cypess et al., 2015). This link between energy balance and AT has gained the interest of researchers over the past few decades as metabolic diseases have become more and more common (Cypess et al., 2015; Kahn et al., 2019).

Adult humans have approximately 50–80 g of active BAT (Shimizu & Walsh, 2015). BAT is composed of multilocular adipocytes which contain small lipid droplets that will only store small amounts of triglycerides within their cells (Smith & Horwitz, 1969). This thermogenically active tissue has been found to contain a specialized protein, uncoupling protein 1 (UCP‐1) that allows it to alter the steps in the electron transport chain to uncouple cellular respiration and ATP synthesis to instead produce heat. Upon cold exposure, the sympathetic nervous system will release norepinephrine which will lead to the activation of BAT thermogenesis, increase the uptake of both glucose and fatty acids, and eventually lead to the production of heat (Cypess et al., 2015; Geer et al., 2014). Due to its ability to expend high amounts of energy, BAT has also been found to contribute to whole‐body metabolism (Shimizu & Walsh, 2015). Numerous studies have linked increased BAT activity to improved glucose metabolism, which has led to the recent interest in further activating this tissue as a possible treatment for metabolic diseases and have been reviewed elsewhere (Bel et al., 2021; Cannon & Nedergaard, 2004; Carpentier et al., 2018; Cypess et al., 2015; Geer et al., 2014; Ramage et al., 2016). While numerous BAT activating agents have been studied, one of the most promising compounds is mirabegron due to its ability to activate the β3‐adrenergic receptors (β3ARs) in AT and increase the metabolic rate of BAT (Bel et al., 2021). The current idea surrounding BAT activation is that increased stimulation of this tissue could help clear glucose and fatty acids and potentially signal as an endocrine organ thereby helping to mitigate metabolic disease.

Metabolic diseases can result from unhealthy AT expansion leading to a range of effects including adipocyte hypertrophy and hyperplasia, the production of pro‐inflammatory cytokines, and increased insulin resistance (Geer et al., 2014; Kahn et al., 2019). These metabolic issues can be produced under chronic exposure to stress hormones and in obesity models that have been shown to impair BAT function and leading to an idea called BAT “whitening” (Shimizu & Walsh, 2015). One of the earliest mentions of what we now call BAT “whitening” is described as the infiltration of WAT into the BAT depot, turning the multilocular BAT adipocytes to a more unilocular appearance (Smith & Horwitz, 1969). Since then, whitening has been linked with obesity, chronic stress, and vascular insufficiencies that will lead to BAT shifting away from performing non‐shivering thermogenesis to act more like a lipid storage organ and contribute to insulin resistance (Glantschnig et al., 2019; Lapa et al., 2017; Luijten et al., 2019; Shimizu & Walsh, 2015). Whitening has been commonly associated with metabolic changes consistent with T2DM (impaired glucose utilization), stress, and metabolic syndrome (Lapa et al., 2017; Shimizu & Walsh, 2015).

Stress causes the release of glucocorticoids (GCs) and is a vital part of physiology (Luijten, Cannon, et al., 2019). GCs are steroid hormones that mediate metabolic changes in order for the animal to respond to stressful stimuli (Bose et al., 2016; Lee et al., 2018). The release of GCs into the circulation is regulated by the hypothalamic–pituitary–adrenal (HPA) axis, which in turn increases the release of stored glucose in the liver (via glyconeogenesis), and stored energy from adipocytes in glycerol and free fatty acids (via lipolysis). This process provides increased substrate availability for ATP synthesis allowing for a burst of energy expenditure in order to escape the stressful situation (Bose et al., 2016; Doig et al., 2017; Glantschnig et al., 2019; Kinlein et al., 2017).

While acute stress is associated with increased immune function and liberation of energy stores, prolonged stress is related to a decrease in immune responses and chronic disease (Cassano et al., 2012; Lee et al., 2018; Luijten, Cannon, et al., 2019). In adipocytes, GCs are needed for mature adipocytes to develop (Kinlein et al., 2017; Lee et al., 2018; Luijten, Cannon, et al., 2019). However, under chronic stress or in metabolic diseases, such as Cushing's disease, this leads to excess GCs being released, where there is increased adiposity, hyperinsulinemia, and decreased glucose tolerance (Kinlein et al., 2017; Luijten, Cannon, et al., 2019).

Corticosterone administered via drinking water is a good model for studying chronic stress‐induced insulin resistance, Cushing's syndrome, hypercortisolemia, dyslipidemia, and influence depressive behavior *in vivo* (Cassano et al., 2012; van Donkelaar et al., 2014; Karatsoreos et al., 2010). There are a variety of factors to consider when implementing this model such as animal age and gender, in addition to the dose and duration of exposure to corticosterone (Kinlein et al., 2017). Younger mice display increased adiposity, blunted growth rates, enhanced glucose clearance, and decreased bone density, which is consistent with the development of Cushing's Syndrome in children (Kinlein et al., 2017). Whereas adult mice displayed increased weight gain, adiposity, and impaired glucose tolerance, consistent with the development of Cushing's as an adult (Kinlein et al., 2017). This investigation aimed at examining the effects of chronic GC treatment with corticosterone in BAT, specifically investigating the effects of whitening and its impact on whole‐body metabolism. Our study explored this effect by exposing young male mice to chronic levels of corticosterone in their drinking water for 4 weeks. We hypothesized that exposure to corticosterone in these mice would result in impaired glucose metabolism, low protein expression levels of UCP‐1, and an overall whitened phenotype in their BAT.

## 2 METHODS

### 2.1 Animals

All protocols were approved by the animal care committee at Lakehead University (AUP 1467374). Four‐week‐old male C57BL/6 (C57BL/6NCrl) mice were purchased from Charles River (St. Constant, Quebec) and allowed to acclimate for 2 weeks prior to treatment. Mice were housed in groups consisting of 3–4 mice per cage at the Pre‐Clinical Research facility. Mice were housed at ambient temperatures (22–25°C) on a 12:12 light–dark cycle with 40–70% humidity. Mice were fed a standard chow diet ad libitum with free access to water.

### 2.2 Treatment preparation and administration

Male mice were exposed to oral treatments (n = 10/treatment) at target doses consisting either of corticosterone (100 µg/ml at the beginning of the treatment and lowered to 50 µg/ml on day 20 of the study), mirabegron (0.0048 or 0.048 mg/ml), vehicle (<1% ethanol), or naïve control (autoclaved water). The dose of 100 µg/ml of oral corticosterone was selected as it was the dose that has been found to model Cushing's disease, metabolic syndrome, and stress‐related obesity (Fransson et al., 2013; Karatsoreos et al., 2010; Tamashiro et al., 2011; Uehara et al., 2020). Mirabegron, a β3AR agonist known to increase BAT activity, was used as a positive control in order to compare the effects of corticosterone against changes observed in BAT under chronic activation. Mirabegron doses were selected based on a study by Sui et al. (2019) who reported BAT activation and WAT beiging under these conditions (Sui et al., 2019). The mirabegron high dose was equivalent to the maximal mirabegron dose administered in humans of 50 mg/day (Nair & Jacob, 2016). Only male mice were used in this study, and this represents a weakness in the study design and an inability to determine the effects of corticosterone in females or a comparison of effects between the sexes. The choice to use only male mice was based primarily on the ability to expand upon previous literature with AT research and adipocyte corticosterone treatment. Most studies using chronic corticosterone as a treatment conducted the experiments in male mice and the differences between the sexes are explained by others (Luine et al., 2017; Rincón‐Cortés et al., 2019). Females also have a heightened stress reactivity when it comes to uncontrollable stress (Rincón‐Cortés et al., 2019). While beiging experiments have been conducted with females, there appears to be some variability in the response to β3AR activation and the beiging response. To successfully understand the effects of our treatment on beiging, males were chosen to be able to determine the use of mirabegron as a beiging agent. However, future studies should be repeated with females in order to understand the treatment effects in both sexes.

All treatments were administered through a water bottle to each group (*n* = 10/treatment) and mice were allowed to drink ad libitum. Corticosterone was purchased from Sigma Aldrich (27840), mirabegron was purchased from Best of Chemical Sciences (B0084‐182334) and 95% ethanol was purchased from Cedarlane (40120791–3).

The appropriate amount of either corticosterone or mirabegron powder was weighed using an analytical balance and stored in canonical tubes until use. Fresh solutions were prepared using the appropriate amount of either corticosterone or mirabegron and ethanol every 3–4 days. Tubes were then placed in a 37°C bead bath for the treatment powders to go into the solution. Treatment solutions were added to autoclaved water to give a final concentration of 100 µg/ml for corticosterone, or 0.0048 mg/ml mirabegron or 0.048 mg/ml mirabegron. Vehicle water was prepared in the manner with ethanol where the final concentration of ethanol in the bottle was <1%. Naïve control water bottles were prepared using autoclaved water. Treatments were administered to the mice through water bottles that were pre‐weighed in order to track water consumption.

The estimated delivered doses of corticosterone and mirabegron were tracked throughout the experiment to ensure the animals received adequate treatment. The corticosterone treated animals started to drink significantly more of the treatment water in the middle of the study, leading to the administered dose being greater than desired. This led to the corticosterone concentration in the treatment water being decreased to 50 µg/ml in order for the animals to receive the targeted dose, after which point the drinking water consumption stabilized. Two corticosterone mice died and were removed from the study; this was likely due to corticosterone‐induced complications, however, this was not tested. One vehicle mouse was removed from the study due to unrelated health complications.

### 2.3 Tissue collection

After 4 weeks of exposure to treatments of either corticosterone or mirabegron, animals were weighed and moved to a clean cage without food (containing water and enrichment) to fast for 5 h prior to dissections. This is a standard procedure for performing metabolic tests of glucose homeostasis in mice (Ayala et al., 2010). Once under the deep surgical plane of anesthesia, blood was collected through cardiac puncture where it was combined with 0.5 M EDTA (pH 8) to give a final mixture of 10% EDTA before the hearts were removed. Blood was mixed by inversion 10 times prior to being stored on ice for a maximum of 2 h before the blood was centrifuged at 3000 × g for 10 min in order to remove plasma and stored at −30°C until use. BAT was dissected within 10 min of cardiac puncture and weighed. The BAT was cut in half, one portion to be used for protein analysis and the other half to be stored in a cassette submerged in formalin for histological analysis. Protein analysis tissues were flash‐frozen in liquid nitrogen and stored on dry ice before long‐term storage at −80°C.

### 2.4 Plasma analysis

Plasma insulin and glucose levels were determined through commercially available kits. Glucose concentrations were determined with a colorimetric assay from Cayman Chemical (1000958) and insulin concentrations were determined with the Mouse Insulin ELISA kit from ALPCO (80‐INSMS‐E01) following the manufacturers’ instructions. The Homeostatic Model of Assessment for Insulin Resistance (HOMA‐IR) was determined for each mouse in the study using the formula: [Fasting Glucose × Fasting Insulin]/405.

### 2.5 Tissue disruption and protein collection

Tissue disruption and protein collection were performed using a modified version of the Removal of Excess Lipids (RELi) protocol described by Marin et al. (2019) (Marin et al., 2019). Fresh RIPA buffer was prepared using 1 M Tris (pH 8), 1.5 M NaCl, 10% NP‐40, 10% Sodium deoxycholate, 10% SDS, and distilled water and stored on ice prior to supplementing with protease inhibitor cocktail (Sigma P8240) (1:500) and phosphatase inhibitor cocktail (1:100) consisting of sodium orthovanadate (1:100) and sodium fluoride (40 µl/ml).

BAT samples were aliquoted with the appropriate amount of RIPA buffer and inhibitors to give a 1:6 weight/volume (300 µl of buffer for every 50 mg of AT) prior to the addition of a metal bead. BAT samples were disrupted at 20 Hz for 2 min, chilled and the process repeated for a total of four times using a Tissue Lyser (Qiagen). The metal bead was removed, and samples were left on ice for 2 h. The samples were centrifuged at 4°C for 15 min at 20,000 × g in order for the supernatant to be separated from the layer of lipids and transferred to a new tube. The process of clearing the supernatants of lipids was repeated three times to reduce the number of lipids in the sample. After the final collection of supernatants, protein quantification of the samples conducted prior to being stored at −80°C.

### 2.6 Protein quantification and sample preparation

Protein quantification was conducted using the Pierce BCA Protein Assay (Thermo Fisher 23227). Samples were diluted 1:3 with 10% SDS and distilled water in order to be utilized in the assay. The manufacturer's instructions were followed using 10 µl of each standard or diluted sample loaded into the plate, in triplicate or duplicate, respectively, prior to the addition of 200 µl of working reagent. For western blots, samples were prepared at a concentration of 0.5 µg/µl in Laemmli sample reducing buffer (125 mM Tris pH 6.8, 2% SDS, 10% glycerol, 0.01% bromophenol blue, and 0.1 M dithiothreitol (DTT), heated at 100°C for 5 min, and then stored at −80°C. The protein concentration for each sample was also determined after being prepped in Laemmli buffer with the Pierce 660 nm assay according to the manufacturer's instructions with the addition of the ionic detergent compatibility reagent (Themo Fisher Scientific PI22660 and PI22663 respectively).

### 2.7 Western blot

Prepared protein samples were separated with 10 or 12% SDS–polyacrylamide gel electrophoresis. 5 µl of BLUelf pre‐stained protein ladder (FroggaBio PM008‐0500) was loaded in the first lane of each gel, followed by an intermembrane control sample and samples from alternating treatment groups. The intermembrane control sample was a prepared BAT sample that was used on each membrane in order to compare the results from each blot back to this sample, thereby allowing for direct comparison across membranes in analysis. Gels were then transferred to either nitrocellulose or PVDF membranes with 30 V for 960 min at 4°C. Membranes were stained with Ponceau S to ensure equal loading before antibody detection. Membranes were blocked for 1 h at room temperature with 5% milk made with 1X TBST. Immunoblotting was performed using either UCP‐1 antibody (Cell Signaling D9D6X) or citrate synthase (ABCAM ab129095) prepared in 5% milk at a dilution of 1:1000 or 1:100,000 respectively. Membranes were incubated in primary antibody solution overnight at 4 °C with agitation. Membranes were washed five times with 1X TBST prior to being incubated in secondary antibody (Goat anti‐rabbit at 1:2500 for UCP‐1 or 1:500,000 for citrate synthase) prepared in 5% milk for 1 h at room temperature. Membranes were then washed again five times with 1X TBST prior to being detected with SuperSignal solution (Thermo Scientific 34577), using the manufacturer's instructions. All blots were quantified using ImageJ software and as stated earlier, the intermembrane control sample was loaded in each gel in order to normalize data between blots. Due to some variability observed in the Ponceau S staining the blots were normalized as follows: the area under the curve from the western blot for the specific protein was normalized to the area under the curve for the intermembrane control used on each gel and then divided by the amount of protein loaded in each lane quantified by the use of the 660 nm assay protein assay.

### 2.8 Histological analysis

Tissues were sent to Ontario Veterinary College at the University of Guelph for slide preparation. Tissues were processed under vacuum using a Shandon Excelsior Tissue Processor. Briefly, the samples were subjected to 70% isopropanol at 30°C for 45 min, 85% isopropanol at 30°C for 45 min, 90% isopropanol at 30°C for 45 min, 95% isopropanol at 30°C for 45 min, 100% isopropanol at 30°C for 45 min twice, Xylene at 30°C for 45 min three times, and Paraffin at 62°C for 45 min three times. Tissues were then embedded with TissuePrep (Fisher Scientific. Cat # T565) and sectioned using a Leica RM255 into 5 µm sections. Slides were stained with hematoxylin and eosin (H&E) in order for histological analysis of the adipocyte area to be conducted. Microscopic images of each BAT were taken at 10X magnification for three separate fields of view for each sample. Analysis of the adipocyte area was conducted using Cell Profiler software as described (Berry et al., 2014) and an example of the how image analysis was conducted for the adipocyte area is depicted in Figure S1.

### 2.9 Statistical analysis

All statistical analyses were performed in GraphPad Prism software. Unless otherwise indicated, means ± SD were calculated for all data sets and displayed in each figure. Data sets were analyzed using one‐way ANOVA with Tukey's post hoc testing, where *p* ≤ 0.05 was considered statistically significant.

## 3 RESULTS

### 3.1 Corticosterone treatment altered mouse physical appearance and drinking water behavior

**FIGURE 1:**
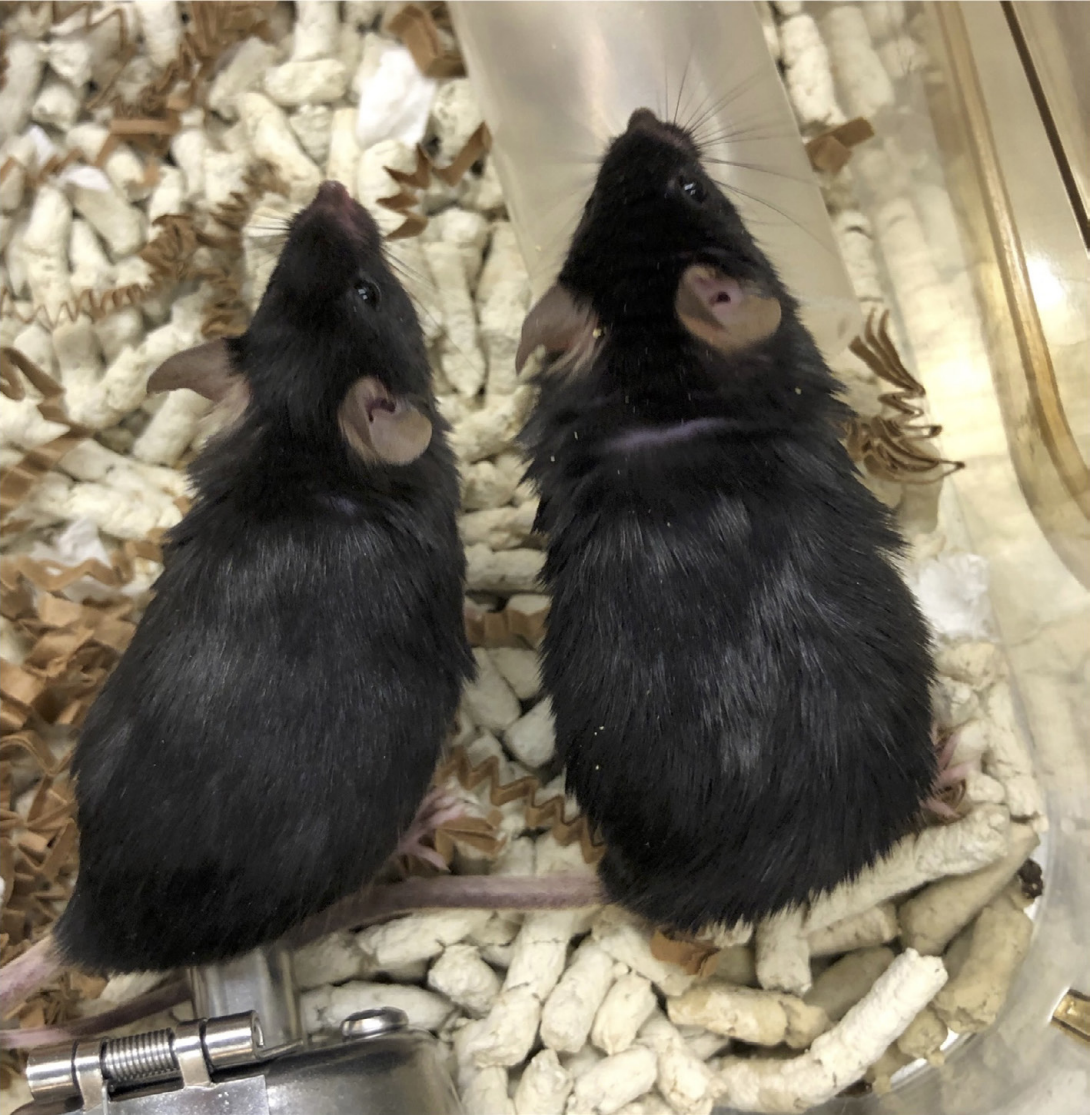
Representative image of the corticosterone‐treated mice. Corticosterone mice appeared to have oily coats

**FIGURE 2:**
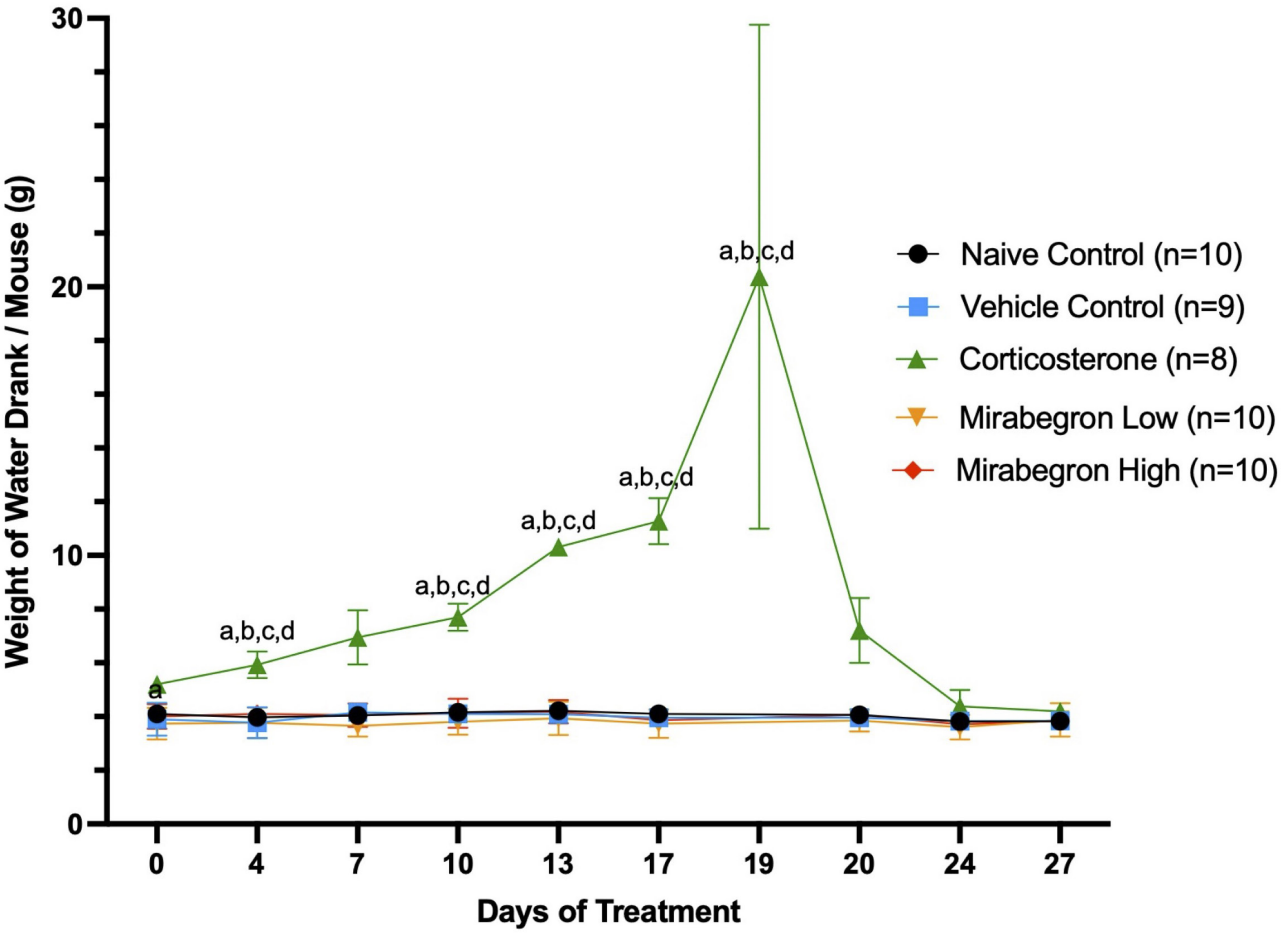
Chronic corticosterone treatment resulted in mice drinking significantly (*p* ≤ 0.05) more of the treatment water during the course of the experiment. (a) represents significantly (*p* ≤ 0.05) different than the naïve control group, (b) represents significantly (*p* ≤ 0.05) different from the vehicle control group, (c) represents significantly (*p* ≤ 0.05) different from the mirabegron low treatment, and (d) represents significantly (*p* ≤ 0.05) different from the mirabegron high treatment

During the course of the experiment chronic treatment with corticosterone altered the overall appearance and drinking water behavior of the mice. Chronic corticosterone treatment resulted in their coats becoming very greasy (displayed in Figure 1). The corticosterone‐treated mice drank significantly (*p* ≤ 0.05) more water than both of the controls and mirabegron treatments beginning on day 10 (Figure 2) (Figures [Link], [Link]). On day 19 of the study, the water consumption increased to the point where the corticosterone concentration needed to be decreased in order to better match the target dose. The corticosterone concentration in the water was decreased to 50 µg/ml from day 20 until the end of the study. At this lower dose, the drinking water behavior became similar to the naïve and vehicle control mice in our study.

### 3.2 Corticosterone treatment increased body and tissue weights

**FIGURE 3:**
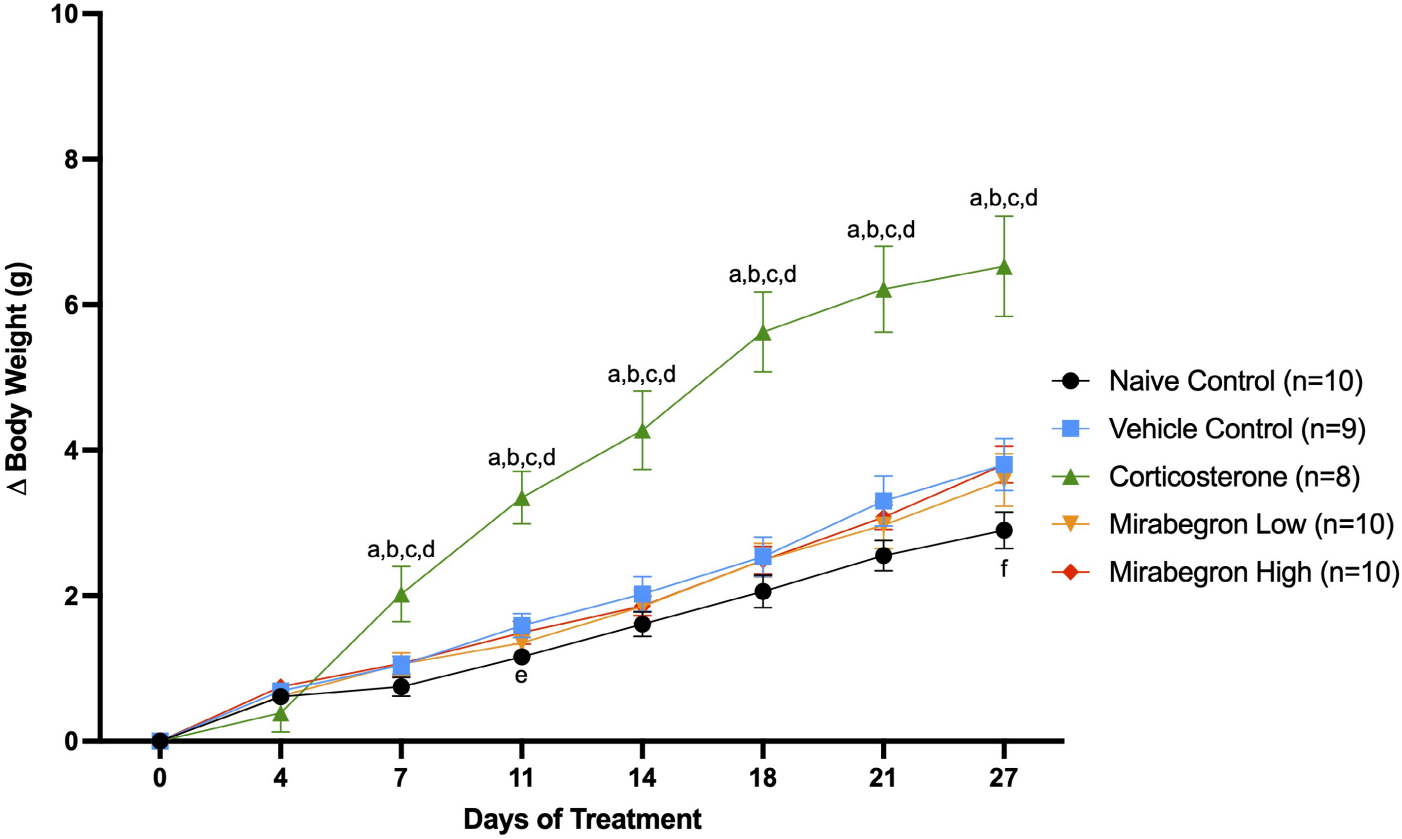
Summary of changes in body weight during four weeks of treatment. The corticosterone‐treated mice gained significantly (*p* ≤ 0.05) more weight than both the naïve and vehicle controls, and both mirabegron treatments. This illustrates the change in body weight from the beginning of the experiment during the treatment. (a) represents significantly (*p* ≤ 0.05) different than the naïve control group, (b) represents significantly (*p* ≤ 0.05) different from the vehicle control group, (c) represents significantly (*p* ≤ 0.05) different from the mirabegron low treatment, (d) represents significantly (*p* ≤ 0.05) different from the mirabegron high treatment, (e) represents the vehicle group being significantly (*p* ≤ 0.05) different than the naïve control group and (f) represents the mirabegron high group being significantly (*p* ≤ 0.05) different than the naïve control group. Error bars represent SEM

**FIGURE 4:**
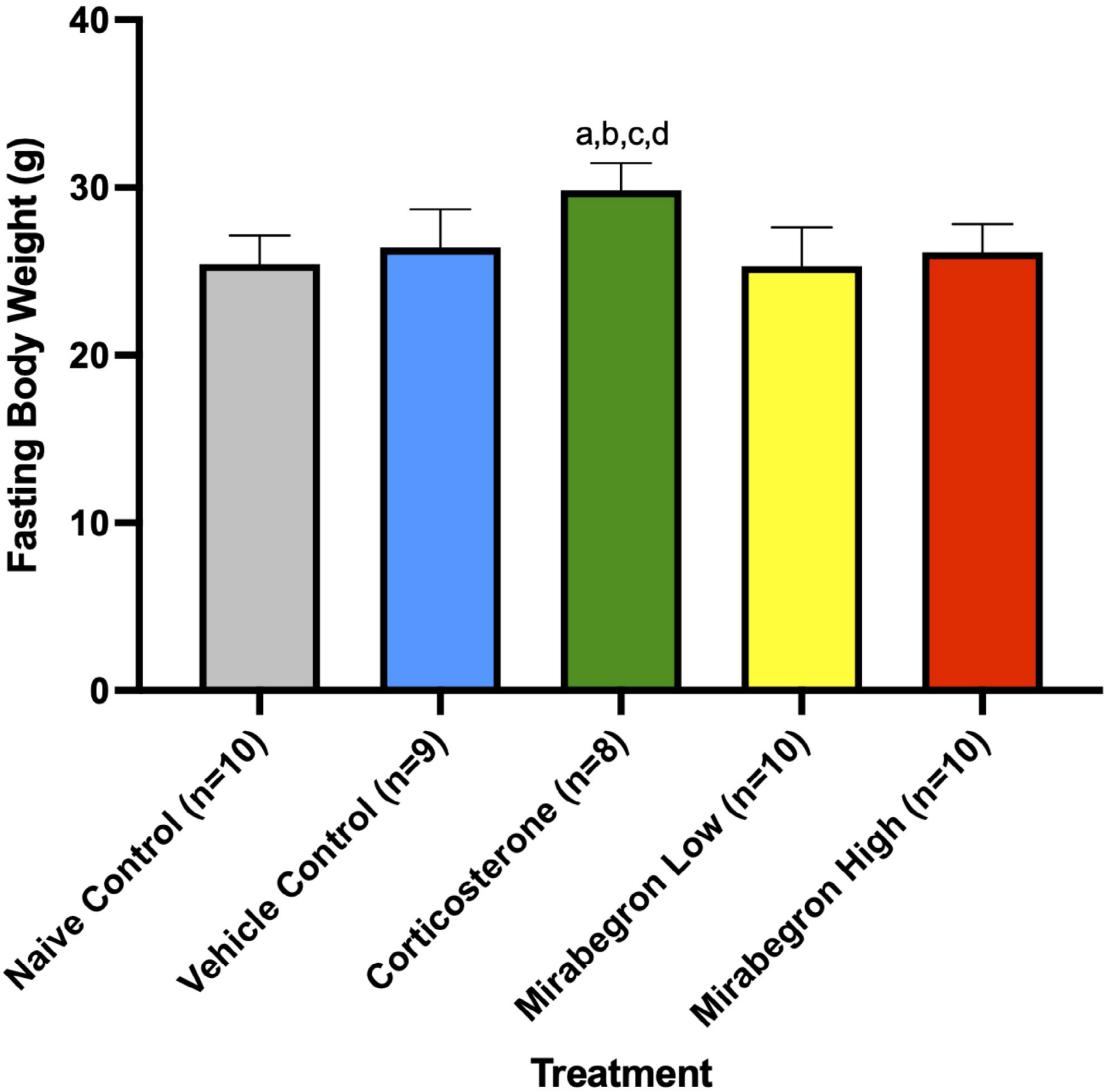
Fasting body weights at the end of treatment. Corticosterone‐treated mice were significantly (*p* ≤ 0.05) heavier than all other treatment groups. (a) represents significantly (*p* ≤ 0.05) different than the naïve control group, (b) represents significantly (*p* ≤ 0.05) different from the vehicle control group, (c) represents significantly (*p* ≤ 0.05) different from the mirabegron low treatment, and (d) represents significantly (*p* ≤ 0.05) different from the mirabegron high treatment

**FIGURE 5:**
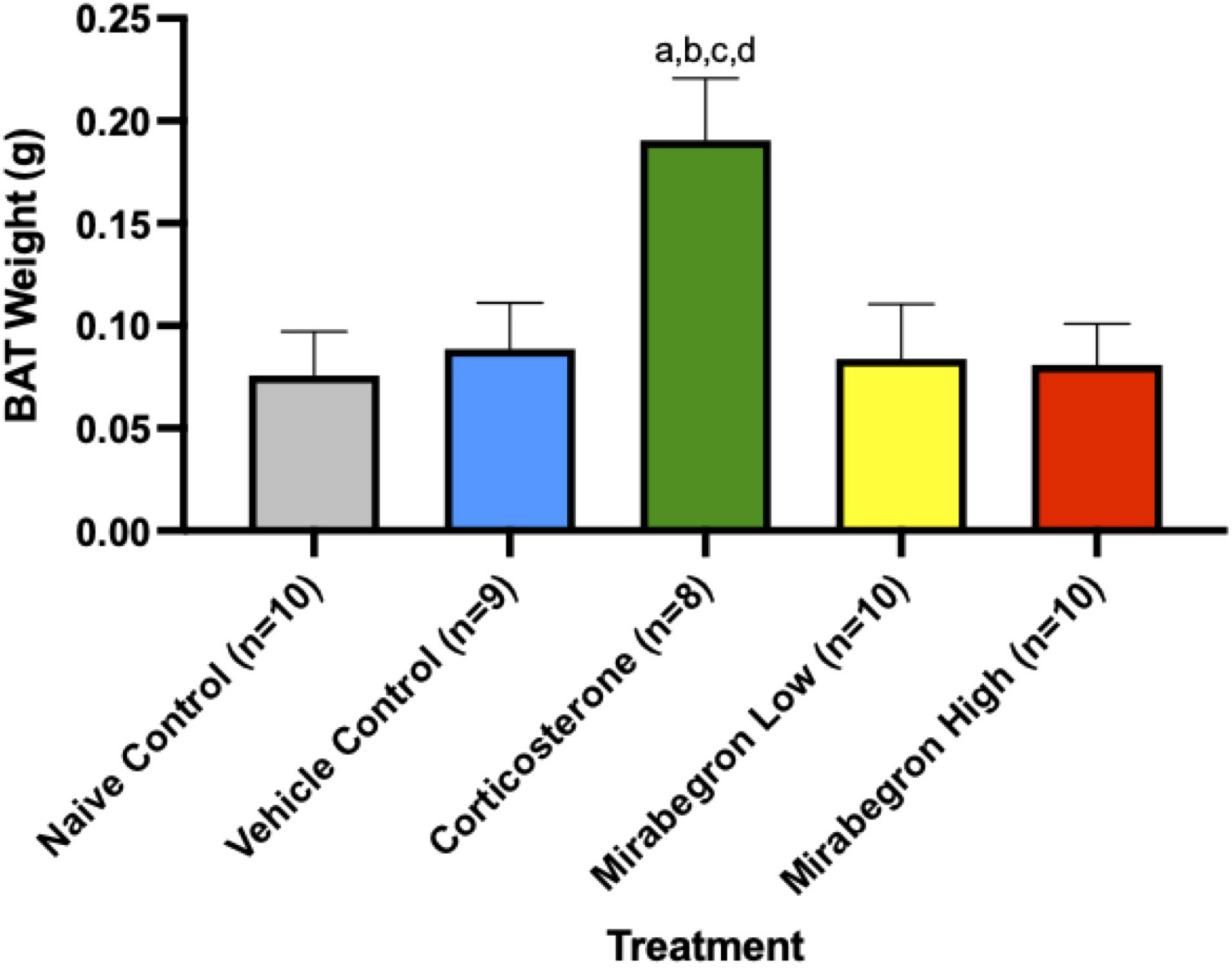
Comparison of the BAT from each treatment. The corticosterone treatment increased BAT weights to be significantly (*p* ≤ 0.05) heavier than all other treatment and control groups in this study. (a) represents significantly (*p* ≤ 0.05) different than the naïve control group, (b) represents significantly (*p* ≤ 0.05) different from the vehicle control group, (c) represents significantly (*p* ≤ 0.05) different from the mirabegron low treatment, and (d) represents significantly (*p* ≤ 0.05) different from the mirabegron high treatment

Throughout the experiment, corticosterone‐treated mice displayed increased body weights (Figure S3) and change in body weight (Figure 3) compared to the naïve and vehicle controls, and both mirabegron doses. Significant increases in fasting body weights (Figure 4), BAT tissue weights alone (Figure 5) and BAT tissue normalized to body weight at the time of tissue collection were also observed. When normalized to body weight, the BAT mass of the corticosterone treated mice remained significantly (*p* ≤ 0.001) higher than both the control and mirabegron treatments (Figure S4).

### 3.3 Corticosterone treatment increased adipocyte area & appearance

**FIGURE 6:**
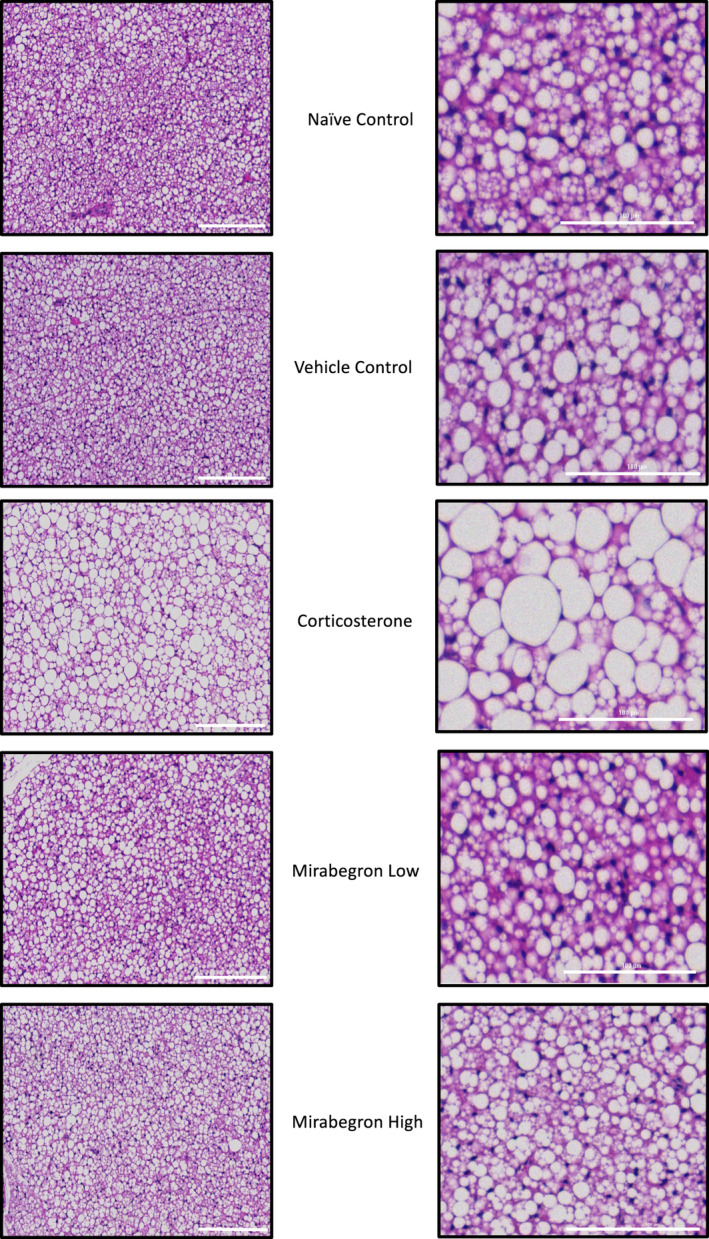
Representative images of BAT at 10× magnification from each treatment where the scalebar represents 200 μm in the large field of view (left) and the magnified image scalebar represents 100 μm (right)

**FIGURE 7:**
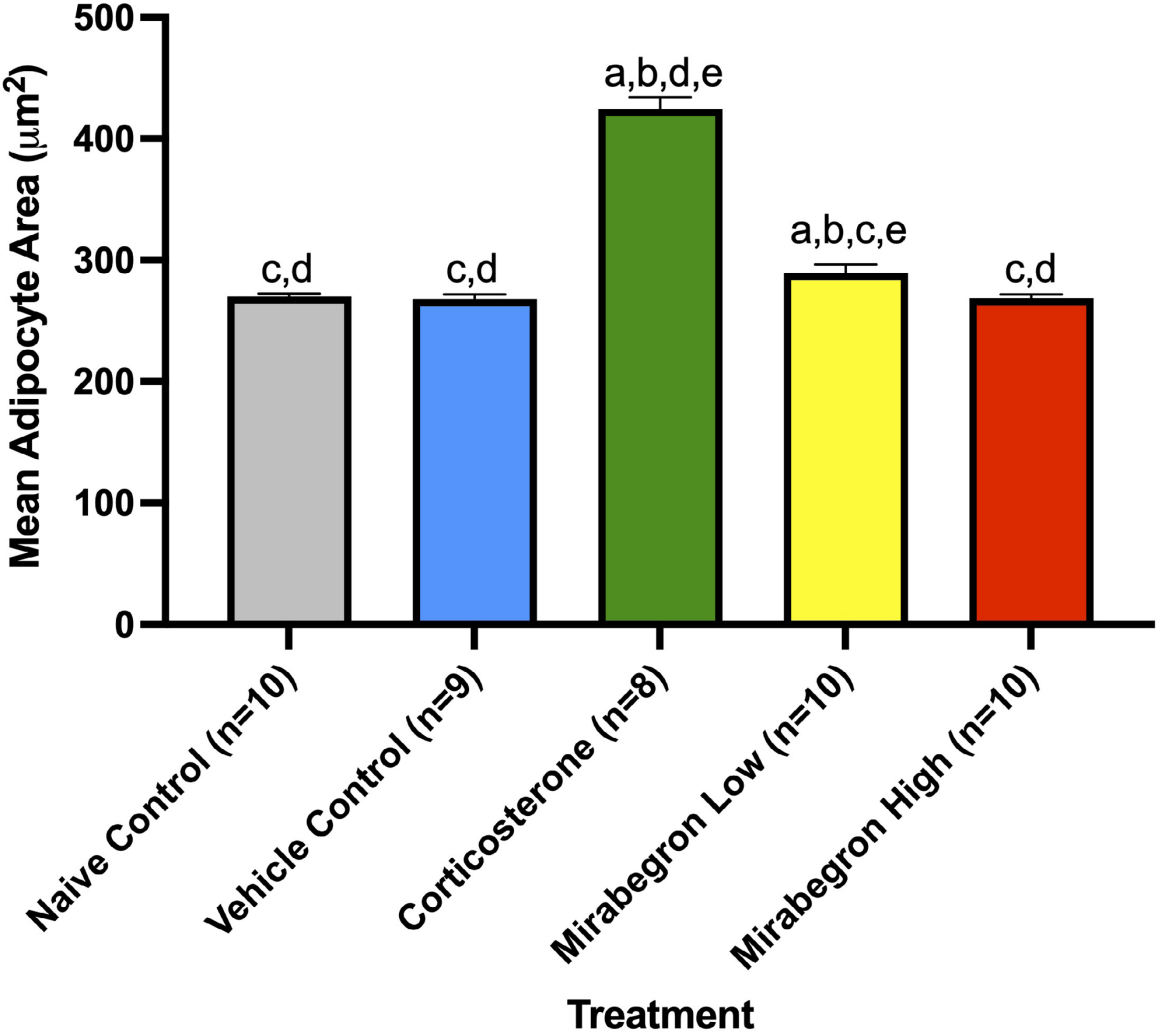
Comparison of mean adipocyte area from each treatment. The corticosterone group had significantly (*p* ≤ 0.05) larger adipocyte areas than all other treatments. (a) represents significantly (*p* ≤ 0.05) different than the naïve control group, (b) represents significantly (*p* ≤ 0.05) different from the vehicle control group, (c) represents significantly (*p* ≤ 0.05) different from the corticosterone treatment, (d) represents significantly (*p* ≤ 0.05) different from the mirabegron low treatment, and (e) represents significantly (*p* ≤ 0.05) different from the mirabegron high treatment

In order to determine the effect of each treatment on adipocyte morphology, histological analysis was conducted to measure BAT adipocyte area. Representative images (Figure 6) and associated analysis (Figure 7) are depicted below. The corticosterone treatment significantly (*p* ≤ 0.05) increased the mean area of the adipocytes (Figure 7) and was accompanied by a pronounced increase in the number of large lipid droplets observed (Figure 6). The adipocyte area of the mirabegron high treatment was similar to the naïve and vehicle controls.

### 3.4 Plasma insulin but not glucose levels were significantly altered by the corticosterone treatment indicating they became insulin resistant

**FIGURE 8:**
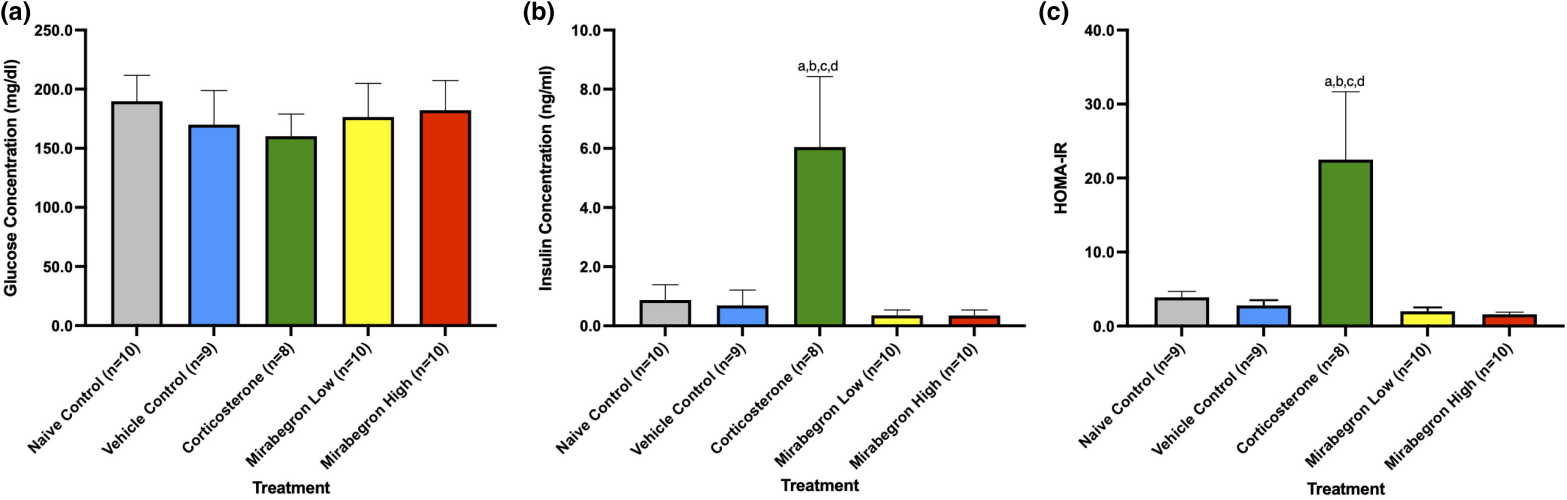
Plasma glucose (a) and insulin (b) measurements, and (c) HOMA‐IR for each treatment. The corticosterone group illustrated significantly (*p* ≤ 0.05) elevated insulin concentrations and insulin resistance (HOMA‐IR) compared to all other treatment groups. (a) represents significantly (*p* ≤ 0.05) different than the naïve control group, (b) represents significantly (*p* ≤ 0.05) different from the vehicle control group, (c) represents significantly (*p* ≤ 0.05) different from the mirabegron low treatment, and (d) represents significantly (*p* ≤ 0.05) different from the mirabegron high treatment

In order to determine the effects of corticosterone or mirabegron on insulin resistance, plasma glucose and insulin concentrations were measured (Figure 8). The HOMA‐IR was determined for each mouse in the study (Figure 8). The corticosterone treatment resulted in less circulating fasting glucose than the controls and mirabegron treatments (Figure 8a). The concentration of fasting insulin in the corticosterone treatment was ~4‐fold greater than the naïve and vehicle controls (*p*‐values 0.0006 and 0.0004 respectively) and both of the mirabegron low and high treatments (*p*‐values 0.0002 and 0.0001 respectively) (Figure 8b). Based on the HOMA‐IR, the corticosterone treatment resulted in insulin resistance that is significantly (*p *≤ 0.05) greater than all other treatments in this study (Figure 8c).

### 3.5 UCP‐1 protein expression was increased by the corticosterone treatment, but citrate synthase protein expression remained unchanged

**FIGURE 9:**
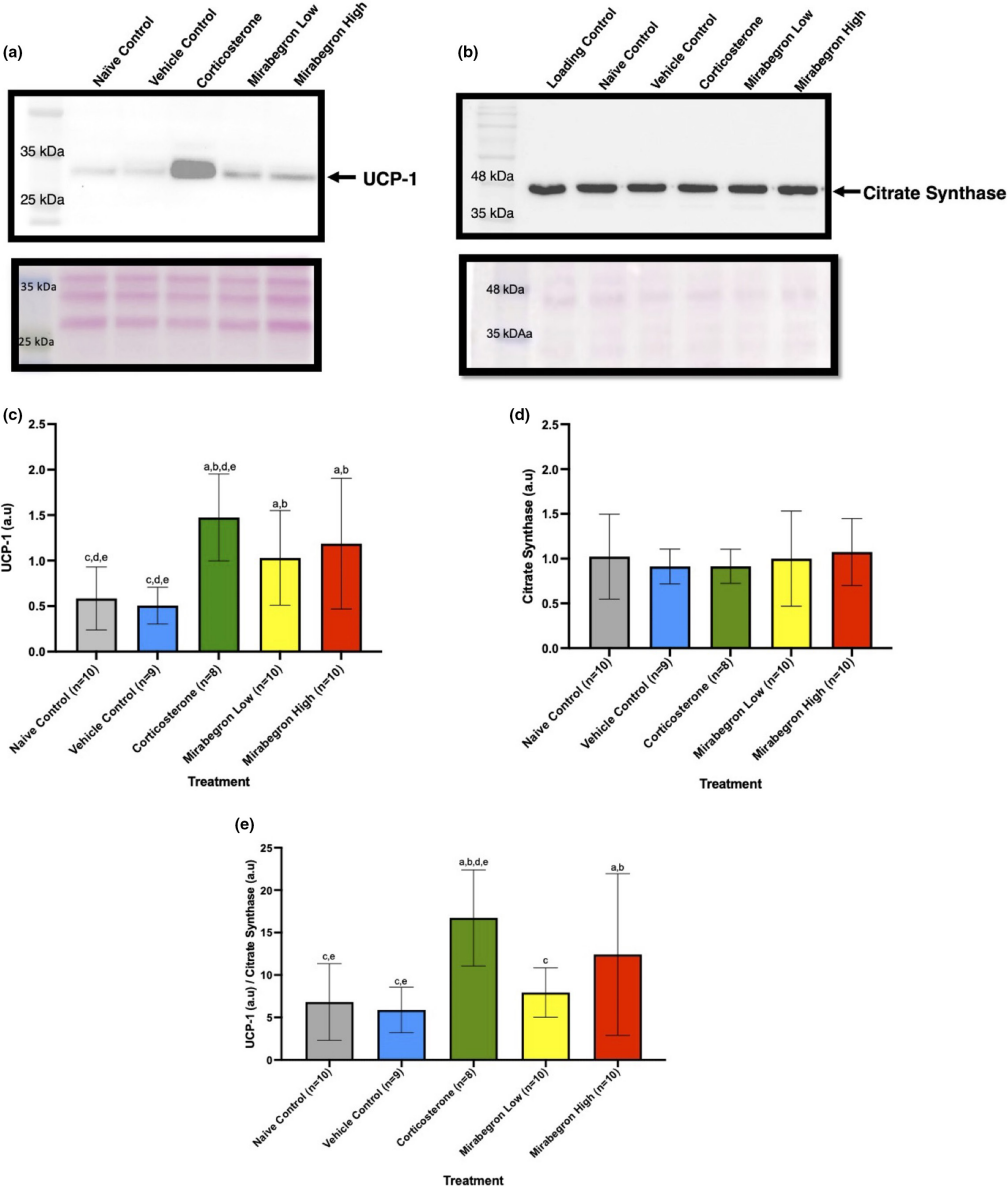
BAT UCP‐1 (a and c) and Citrate Synthase (b and d) protein expression under each treatment and relative ratio (e). The corticosterone group illustrated a significant (*p* ≤ 0.05) increase in the amount of UCP‐1 (a and c) expressed in each BAT when compared to the naïve and vehicle control groups (corresponding ponceau S stain is below the blot). The citrate synthase protein expressed (b and d) in each BAT remained the same amongst all treatments (corresponding ponceau S stain is below the blot). The observed size of citrate synthase is 45 kDa while the protein size is 51 kDa according to the manufacturer. The ratio of UCP‐1 to citrate synthase (e) is significantly (*p* ≤ 0.05) greater in both the corticosterone and mirabegron high treatments compared to both the naïve and vehicle controls. (a) represents significantly (*p* ≤ 0.05) different than the naïve control group, (b) represents significantly (*p* ≤ 0.05) different from the vehicle control group, (c) represents significantly (*p* ≤ 0.05) different from the corticosterone treatment, (d) represents significantly (*p* ≤ 0.05) different from the mirabegron low treatment, and (e) represents significantly (*p* ≤ 0.05) different from the mirabegron high treatment

Western blot analysis was performed in order to measure the protein expression of both UCP‐1 and citrate synthase under each treatment. The chronic corticosterone treatment significantly (*p* ≤ 0.05) increased the UCP‐1 expression in the BAT depot (Figure 9). This increased UCP‐1 protein expression was not likely the result of increased mitochondrial content, as the protein expression of citrate synthase remained constant amongst the treatment groups (Figure 9). Mirabegron treatment resulted in increased UCP‐1 expression at both the low and high doses were significantly greater than the naïve (*p*‐values 0.0476 and 0.0085 respectively) and vehicle controls (*p*‐values 0.0246 and 0.0041 respectively). When comparing the relative protein expression of UCP‐1 to citrate synthase, there was a significant (*p* ≤ 0.05) increase in the level of UCP‐1 in both the corticosterone treatment (*p*‐values 0.0009 and 0.0004) and the mirabegron high treatment groups (*p*‐values 0.0370 and 0.0185) compared to both naïve and vehicle controls respectively (Figure 9).

## 4 DISCUSSION

In order to examine the effects of whitening and whole‐body metabolism in BAT we exposed young male mice to chronic levels of corticosterone in their drinking water for 4 weeks. We hypothesized that these mice would have impaired glucose metabolism, low protein expression levels of UCP‐1, and an overall whitened phenotype in their BAT. To our knowledge, this is the first study to report significantly (*p* ≤ 0.05) increased UCP‐1 protein expression accompanying a whitened phenotype in BAT under chronic treatment with corticosterone in just 4 weeks. Previous studies with GC treatments in both rodents and humans report decreased BAT activation, decreased UCP‐1 mRNA and protein levels, in combination with whitened BAT and whole‐body insulin resistance (van den Beukel et al., 2015; Poggioli et al., 2013; Ramage et al., 2016; Strack et al., 1995; Thuzar et al., 2018).

Administration of corticosterone via drinking water has been used as a model for metabolic syndrome, chronic stress‐induced T2DM, hypercortisolemia, dyslipidemia, obesity and Cushing's Syndrome by many others, (Cassano et al., 2012; Do et al., 2019; van Donkelaar et al., 2014; Karatsoreos et al., 2010). This model has also been described by Gasparini et al. (2016) to mimic clinical GC therapy and was the first study to directly compare corticosterone administration models *in vivo* (Gasparini et al., 2016). Gasparini et al. (2016) directly compared corticosterone administered via drinking water to the pellet implantation method and their results further validated the use of drinking water to expose mice to excess GCs (Gasparini et al., 2016). Using drinking water to administer corticosterone has been found to be the least invasive to the animal and its diurnal rhythm while also allowing for the effects of long‐term stress to be studied in a rapid and safe manner (Cassano et al., 2012; van Donkelaar et al., 2014; Gasparini et al., 2016; Karatsoreos et al., 2010). Even though this model has been extensively used, our findings represent a novel report based on the collection of findings. Chronic corticosterone treatment altered the coat of the animals to become oily/greasy in appearance (Figure 1). Although we did not directly measure home cage locomotion, these corticosterone treated mice also appeared to be less active than our other treatment groups. This decreased locomotion has been previously described in corticosterone‐treated mice (Cassano et al., 2012; Do et al., 2019; Karatsoreos et al., 2010). van Donkelaar et al. (2014) also measured the relative distance moved in corticosterone‐treated mice and found that this treatment results in less distance covered compared to vehicle controls (van Donkelaar et al., 2014).

Many previous studies using corticosterone in drinking water have not reported dramatic increases in the volume of water consumed. The corticosterone‐treated mice drank significantly (*p* ≤ 0.05) more water than any of the naïve or vehicle controls and both mirabegron treatments during the course of the study (Figure 2). While this did alter the dose of the treatment they received (Figure S2), it was corrected after day 20 of the experiment. Once lowered to a dosage of 50 µg/ml, the drinking behavior became similar to naïve and vehicle controls (Figure 2), although the final 8 days resulted in a slightly lower dose than what was targeted (Figure S2). Studies conducted previously using the same dose, delivery mode, and housing temperatures did not widely report this increased water intake or the dose of corticosterone actually consumed by the mice (van Donkelaar et al., 2014; Karatsoreos et al., 2010). Only a handful of studies have reported a significant increase in water intake at this dose and housing temperature, and Cassano et al. (2012) and Kinlein et al. (2017) reported this increase began from 14 days of exposure, similar to our study (Cassano et al., 2012; Do et al., 2019; Kinlein et al., 2017; Luijten, Brooks, et al., 2019). Plasma corticosterone levels were reported by two of these studies, but none have reported the actual dose of corticosterone the animals received based on water consumption (Do et al., 2019; Kinlein et al., 2017). One study conducted was conducted at both thermoneutral conditions (30°C) and at 21°C reported increased water intake in both groups of corticosterone‐treated mice (Luijten, Brooks, et al., 2019). With corticosterone treated mice illustrating such an increase in drinking water volume, future studies should alter the dose administered accordingly and include either intermediate or dose‐corrections to account for this increased water consumption.

In the present study, fasting body and BAT weights of the corticosterone treated mice were significantly (*p* ≤ 0.05) increased compared to all other treatments (Figure 3) (Figure 4) (Figure S3) (Figure 5) (Figure S4). This increase in body weight in the corticosterone treated mice compared to controls is in agreement with other studies (Do et al., 2019; van Donkelaar et al., 2014; Karatsoreos et al., 2010; Kinlein et al., 2017). In contrast, chronic BAT activation with mirabegron did not alter BAT mass. Increased body weight is a hallmark of GC excess and illustrates the body's metabolic shift that occurs under these conditions. Other groups have also reported increased gonadal and overall white AT under chronic corticosterone treatment (Gasparini et al., 2016; Karatsoreos et al., 2010), with one study specifically reporting an increase in BAT weight under these standard housing and corticosterone treatment conditions for 2‐weeks (Luijten, Brooks, et al., 2019). Do et al. (2019) conducted a similar study but treated mice for 8‐weeks. They reported increased body and BAT mass that remained significant throughout 8‐weeks of corticosterone exposure (Do et al., 2019). This illustrates that our model of chronic exposure to GCs reveals the same AT mass trend as longer duration studies.

After 4 weeks of corticosterone treatment, the average adipocyte area was significantly (*p* ≤ 0.05) increased compared to both the naïve and vehicle control groups and both mirabegron treatments (Figures 6, 7). This increased adipocyte size was not the result of chronic activation of the β3AR pathway as the mirabegron treated groups did not alter the adipocyte areas. This larger adipocyte area is representative of the BAT undergoing “whitening” where it is shifting its phenotype to store lipids. This adipocyte expansion has been found under both standard and thermoneutral housing conditions in both mouse and rat models (Cassano et al., 2012; Luijten, Brooks, et al., 2019; Mousovich‐Neto et al., 2019). Luijten, Cannon, et al. (2019) directly compared the adipocyte size of mice exposed to chronic corticosterone treatments housed both at thermoneutrality and at standard housing temperatures and found that corticosterone treatment resulted in increased adipocyte/lipid droplet area regardless of room temperature (Luijten, Brooks, et al., 2019). This histological shift further exemplifies the whole‐body response changing under GC excess. While this study aimed at investigating the effects of chronic treatment with corticosterone, acute treatment at room temperature with corticosterone pellets (50 mg) being implanted in mice for 7 days has been investigated (van den Beukel et al., 2015). The HPA axis activity of the corticosterone treated mice were reduced, they had increased plasma triglyceride concentrations, reduced BAT activity, and increased BAT weight (van den Beukel et al., 2015). These findings illustrate that corticosterone elicits a strong effect on the body and induces alterations in as little as 7 days.

Impaired glucose clearance is a key component of metabolic syndromes and was described in chronic corticosterone‐treated animals in as little as 4 weeks (when performing a glucose tolerance test) (Karatsoreos et al., 2010; Kinlein et al., 2017). While our study did not measure oral glucose tolerance, we observed a decrease in plasma glucose concentrations compared to naïve control mice (Figure 8a). Other studies with corticosterone treatment administered via drinking water reported increased glucose concentrations in corticosterone‐treated mice, however, this increase was found to occur after 5 weeks of treatment which may account for why we did not observe this trend in our experiment (Burke et al., 2017; Cassano et al., 2012). Kinlein et al. (2017) compared the effects of corticosterone treatments in adolescent (3 weeks) and adult (>8 weeks) mice (Kinlein et al., 2017). While the findings with glucose tolerance in adult mice match what has been previously reported, adolescent mice display lower plasma glucose levels following glucose challenges at the 15 and 30‐minute time points (Kinlein et al., 2017). This lower plasma glucose level shows some degree of enhanced glucose clearance, similar to what was observed in our study under fasting conditions in young mice, which may be related to the increased fasting insulin levels observed (Figure 8b). Given the different results in fasting glucose in adult (62 days old) vs adolescent (22 days old) mice, there may be an age effect on the glucose metabolism under chronic corticosterone treatment that cannot be ignored (Kinlein et al., 2017).

Fasting insulin concentrations were significantly (*p* ≤ 0.05) greater in the corticosterone‐treated mice, compared to controls and mirabegron treated groups in this study (Figure 8b). GC‐induced insulin resistance has been reported in corticosterone‐treated mice at this dose and housing temperature (van Donkelaar et al., 2014; Gasparini et al., 2016; Karatsoreos et al., 2010; Kinlein et al., 2017). Using the HOMA‐IR, we were able to determine that corticosterone treatment for 4 weeks results in severe insulin resistance (Figure 8c). Other researchers have also noted that this level of insulin resistance remains, even when corticosterone treatment lasts for 12‐weeks (van Donkelaar et al., 2014). Burke et al. (2017) explained the development of insulin resistance as the reciprocal relationship between body composition with a decrease in lean mass and an increase in fat mass (Burke et al., 2017). This alteration in insulin sensitivity with corticosterone exposure has also been linked to increased expressions of genes promoting glucose metabolism (Burke et al., 2017) and protein breakdown in the muscle (Burke et al., 2017; Kinlein et al., 2017). Karatsoresos et al. (2010) reported elevated plasma leptin and insulin levels under corticosterone treatment, and these two measurements were correlated with one another (although these measurements were conducted on animals who were in the fed state) (Karatsoreos et al., 2010). Karatsoreos et al. (2010) connected the increased plasma leptin, high levels of WAT, and increased hyperphagia to leptin resistance (Karatsoreos et al., 2010). Due to leptin's origin being the adipocytes, it is a logical connection between leptin resistance and high levels of WAT (Karatsoreos et al., 2010). Chronic treatment with corticosterone was found to be equivalent to those found in diet‐induced obese mice, connecting the importance of leptin and obesity to AT (Karatsoreos et al., 2010). The exact mechanism of GC‐induced changes on leptin and insulin remains unclear, but more studies are beginning to hint at these answers.

Linking GCs and BAT thermogenesis has been previously conducted in healthy lean men, where acute administration of prednisone acutely increased BAT ^18^F‐FDG uptake upon mild cold exposure (Ramage et al., 2016). In vitro analysis of cells from these men also revealed an increase in UCP‐1 protein expression in the BAT tissue (Ramage et al., 2016). However, a double‐blind study with men and women exposed to prednisone and subsequent cold exposures revealed that the GC suppressed BAT activity after one week (Thuzar et al., 2018). This link between activation of UCP‐1 and GCs brings up further questions into how UCP‐1 is exactly expressed when most rodent studies report decreased UCP‐1 expression with GCs. It has been suggested that GCs result in BAT gaining a “pseudo‐atrophy” state where it will exhibit large lipid storage capabilities but can also sustain some of its thermogenic capacity (Luijten, Cannon, et al., 2019). This is a deviation from the conventional understanding that GCs will induce excess lipid accumulation in BAT and the obese state will greatly lower the tissue's thermogenic capacity (Beaupere et al., 2021; Lee et al., 2014; Luijten, Cannon, et al., 2019; Reddy et al., 2014). Western blot analysis of BAT protein revealed that the chronic corticosterone treatment significantly (*p* ≤ 0.05) increased UCP‐1 protein expression (Figure 9a). This chronic glucocorticoid treatment even displayed higher levels of BAT UCP‐1 protein expression than the mirabegron treatments, which directly target the β3AR receptor (Figure 9a and c). In order to determine if the mitochondrial content of these BAT depots were altered and thereby giving this unexpected result, we also measured citrate synthase protein levels in each sample as it is a biomarker for mitochondrial content (Larsen et al., 2012). As depicted in Figure 9b, the amount of this protein was not different between sample groups. This represents an interesting finding in that mitochondrial content did not change even though UCP‐1 expression increased in corticosterone and both mirabegron treatments. Variations in citrate synthase expression were found in a clinical trial investigating male omental AT where this protein displayed lower expression levels in the obese and higher protein levels in the diabetic patients (Christe et al., 2013). When comparing the ratio of UCP‐1 to citrate synthase, Figure 9c illustrates that the corticosterone treated mice have significantly (*p* ≤ 0.05) higher relative UCP‐1 levels than both naïve and vehicle control groups and the mirabegron low treatment. The mirabegron high treatment also displays significantly (*p* ≤ 0.05) higher relative UCP‐1 than the naïve and vehicle control groups (Figure 9). The increased relative UCP‐1 in the mirabegron high treatment was not a surprise as this β3AR agonist functions to increase the BAT activation in this manner. However, the increased level of UCP‐1 per mitochondria in the corticosterone treated mice is a novel finding. As noted earlier, this relationship is a deviation from the conventional understanding that GCs will induce lipid accumulation in BAT and this obese state will lower the tissue's thermogenic capacity (Beaupere et al., 2021; Lee et al., 2014; Luijten, Cannon, et al., 2019; Reddy et al., 2014). However, a further examination into obesity models reveals a plausible explanation. A study conducted at thermoneutrality (30°C) for a 12‐week treatment of 50 µg/ml corticosterone in drinking water also studied the effects of GC‐induced obesity and its effects on UCP‐1 (Luijten, Brooks, et al., 2019). Their treatment also caused severe obesity, however, UCP‐1 expression was only decreased under thermoneutral conditions, not under standard housing temperatures (Luijten, Brooks, et al., 2019). Luijten, Brooks, et al. (2019) further raise the question as to how GCs channel energy from food into lipid storage, as they also found GC‐induced obesity in UCP‐1 knockout (KO) mice (Luijten, Brooks, et al., 2019). These researchers also discuss a notable concept related to examining UCP‐1 protein levels and making conclusions about its effect on physiological significance. They conclude that the UCP‐1 expression should be reported as UCP‐1 per BAT protein, as this will represent physiological significance when it comes to thermogenic capacity (Luijten, Brooks, et al., 2019). When comparing total protein levels from BAT of corticosterone‐treated mice, Luijten, Brooks, et al. (2019) observed a decrease in the total amount of protein extracted when mice were housed at thermoneutrality (Luijten, Brooks, et al., 2019). Luijten, Brooks, et al. (2019) investigated the effects corticosterone had on UCP‐1 and reported that brown adipocytes isolated from corticosterone treated mice responded with increased oxygen consumption to an artificial uncoupler, indicating that their result of lower norepinephrine‐induced oxygen consumption was not the result of decreased respiratory chain capacity (Luijten, Brooks, et al., 2019). Moreover, corticosterone‐treated mice at standard housing conditions had a reduction in their underlying metabolism accompanied by increased food intake, both of which contributed to their obese state (Luijten, Brooks, et al., 2019). Under standard housing conditions, they found no effect on total BAT UCP‐1, or total thermogenic capacity, even though corticosterone treatment‐induced obesity in these mice (Luijten, Brooks, et al., 2019). Luijten, Brooks, et al. (2019) also concluded that at thermoneutrality, the total amount of BAT UCP‐1 protein is not influenced by the corticosterone‐induced AT expansion (Luijten, Brooks, et al., 2019). Although the increase in AT mass increased from week two until the end of treatment (week 12), their body mass stabilized in the obese state and the effects on UCP‐1 protein levels were not maintained as they too normalized in the BAT (Luijten, Brooks, et al., 2019). When exposed for 12‐weeks at thermoneutrality, Luijten, Cannon, et al. (2019) reported that corticosterone‐treated animals displayed increased energy expenditure in the light phase and this led to an increase in norepinephrine‐induced oxygen consumption (Luijten, Brooks, et al., 2019). Doig et al. (2017) reported that 11β‐HSD1 KO mice displayed increased UCP‐1 protein expression and attributed this increase as a way for BAT to become more sensitive to GCs, and therefore acting as a potential mechanism for efficient thermogenic activity (Doig et al., 2017).

Diet‐induced thermogenesis (DIT) has been found to increase UCP‐1 under an obesogenic diet (containing high amounts of fat) (von Essen et al., 2017). However, von Essen et al. (2017) report that the total amount of UCP‐1 protein at thermoneutrality is dependent on the total BAT content itself (von Essen et al., 2017). When DIT occurs, the more UCP‐1 present in BAT may limit obesity development during the HFD exposure, but it will not prevent obesity (von Essen et al., 2017). Alcala et al. (2017) conducted studies on HFD obese mice specifically to investigate BAT and WAT dysfunction (Alcala et al., 2017). This study notably reported increased UCP‐1 protein expression without altering the mitochondrial content of BAT (Alcala et al., 2017). This increased UCP‐1 protein content was not reflected in the mRNA levels of BAT (Alcala et al., 2017). The BAT from these obese mice had increased mRNA levels of endoplasmic reticulum (ER) stress and pro‐inflammatory markers, but protein levels were not altered (Alcala et al., 2017). The obese BAT also illustrated higher reactive oxygen species (ROS) levels and enhanced mitochondrial respiration (2X as much as lean BAT), illustrating that this tissue responds to obesity very differently than what was conventionally understood (Alcala et al., 2017). The initial increase in inflammation, ER stress, and generation of ROS was not high enough to completely alter the fuel burning capacity of the tissue (Alcala et al., 2017). Alcala et al. (2017) explained the increased UCP‐1 protein content to arise from the accumulation of protons in the intermembrane space being pushed back into the mitochondrial matrix and from the free fatty acids activating the protein itself, further promoting its fuel‐burning capabilities (Alcala et al., 2017). Findings from a recent study involving obese humans found an increase in *Ucp*‐*1* mRNA expression in the visceral AT in conjunction with increased resting energy expenditure (Bettini et al., 2019). The authors of this study conclude that the increase in *Ucp*‐*1* could represent a mechanism in which the AT is attempting to further prevent weight gain in these obese individuals (Bettini et al., 2019). The idea that increased uncoupling can result from the tissue increasing its energy‐burning ability in order to respond to inflammation, ER stress, and ROS could help explain why we found increased UCP‐1 expression under chronic corticosterone treatment. The higher dose of corticosterone received as a result of the increased water consumption likely played a key role in initiating these alterations in the BAT that have not been previously observed by others. However, in combination with the studies from Alcala et al. (2017) and Bettini et al. (2019), we can speculate that BAT may exhibit a compensatory mechanism that can increase the fuel‐burning capabilities through UCP‐1 when presented with chronic lipid accumulation. Increased protein expression of UCP‐1 at room temperature under both significantly high doses of corticosterone (observed in our study) or in cases of severe obesity illustrates that there may be more dynamic pathways involved in the BATs ability to respond to increased lipid storage than what is currently understood. We know that the increased UCP‐1 was higher than the mirabegron treated mice which means that the corticosterone treatment activated some other pathway than through the β3ARs.

Our data are the first to demonstrate increased UCP‐1 within GC‐induced whitened BAT. In combination with the findings reported by Alcala et al. (2017) of obese BAT displaying enhanced mitochondrial respiration, we illustrate that chronic exposure to corticosterone induces insulin resistance, increased whole‐body and BAT mass, increased adipocyte area, and increased UCP‐1 protein expression, while the mitochondrial content of the tissue remains unchanged. This increased uncoupling potential could be a compensatory mechanism being undertaken by the tissue for excess caloric intake as lipid storage increases dramatically. Increased UCP‐1 protein expression has been widely regarded as a beiging phenotype accompanied by improved glucose and insulin sensitivities, as displayed through the mirabegron treatments in this study. However, the same level of increased UCP‐1 protein expression found in a whitened BAT requires further investigations into this alternative uncoupling pathway. Future studies investigating excess GCs and BAT uncoupling potential is fundamental to our understanding of this tissue and could provide future targets to explore when treating metabolic diseases. They should also investigate insulin signaling in BAT in these treatments to fully understand the extent of their impact on glucose metabolism.

### 4.1 Limitations of the study

One weakness of our study is that it was limited to only male mice and did not allow for the investigation into females or the comparison between sexes. While this decision was made for a variety of reasons discussed earlier, future studies should include females in order to understand the effects of these treatments in both sexes.

As discussed above, we encountered a challenge with maintaining our targeted dose of corticosterone. The corticosterone mice started drinking significantly (*p* ≤ 0.05) more of the treatment water during the course of our study which resulted in the mice receiving a dose that was greater than our target. While we did correct for this by lowering the concentration in the corticosterone treatment water, this may have impacted our results.

### 4.2 Conclusions

Our *in vivo* model illustrated that chronic treatment with corticosterone resulted in an altered phenotype, increased insulin resistance, induced BAT tissue remodeling and whitening, while also significantly (*p* ≤ 0.05) activating thermogenic protein UCP‐1 and uncoupling, possibly indicating a protective mechanism is being initiated for mitigating excess energy loads. To our knowledge, this is the first study to illustrate this relationship under chronic stress, namely with increased UCP‐1 expressions in BAT, indicating that UCP‐1 may be more involved in the stress response than what is currently known.

## ETHICS STATEMENT

All animal experimental and care procedures were performed in accordance with the guidelines of the Canadian Council on Animal Care and approved by the Animal Care Committee of Lakehead University.

## AUTHOR CONTRIBUTIONS

Jocelyn S. Bel, Simon J. Lees, Neelam Khaper, and T.C. Tai conceived the ideas in the manuscript. JSB performed the experiments, curated the data, and prepared the manuscript. Simon J. Lees, Neelam Khaper, and T.C. Tai critically revised the article for important intellectual content and flow. All authors approved the final version of the manuscript and agree to be accountable for all aspects of the work in ensuring that questions related to the accuracy or integrity of any part of the work are appropriately investigated and resolved.

## Supporting information



Fig S1

Fig S2

Fig S3

Fig S4
