# Supplementary figures and images for: Chronic glucocorticoid exposure causes brown adipose tissue whitening, alters whole‐body glucose metabolism and increases tissue uncoupling protein‐1
Source: Physiol Rep. 2022 May 4;10(9):e15292. doi: 10.14814/phy2.15292 (PMC9069169; doi:10.14814/phy2.15292)

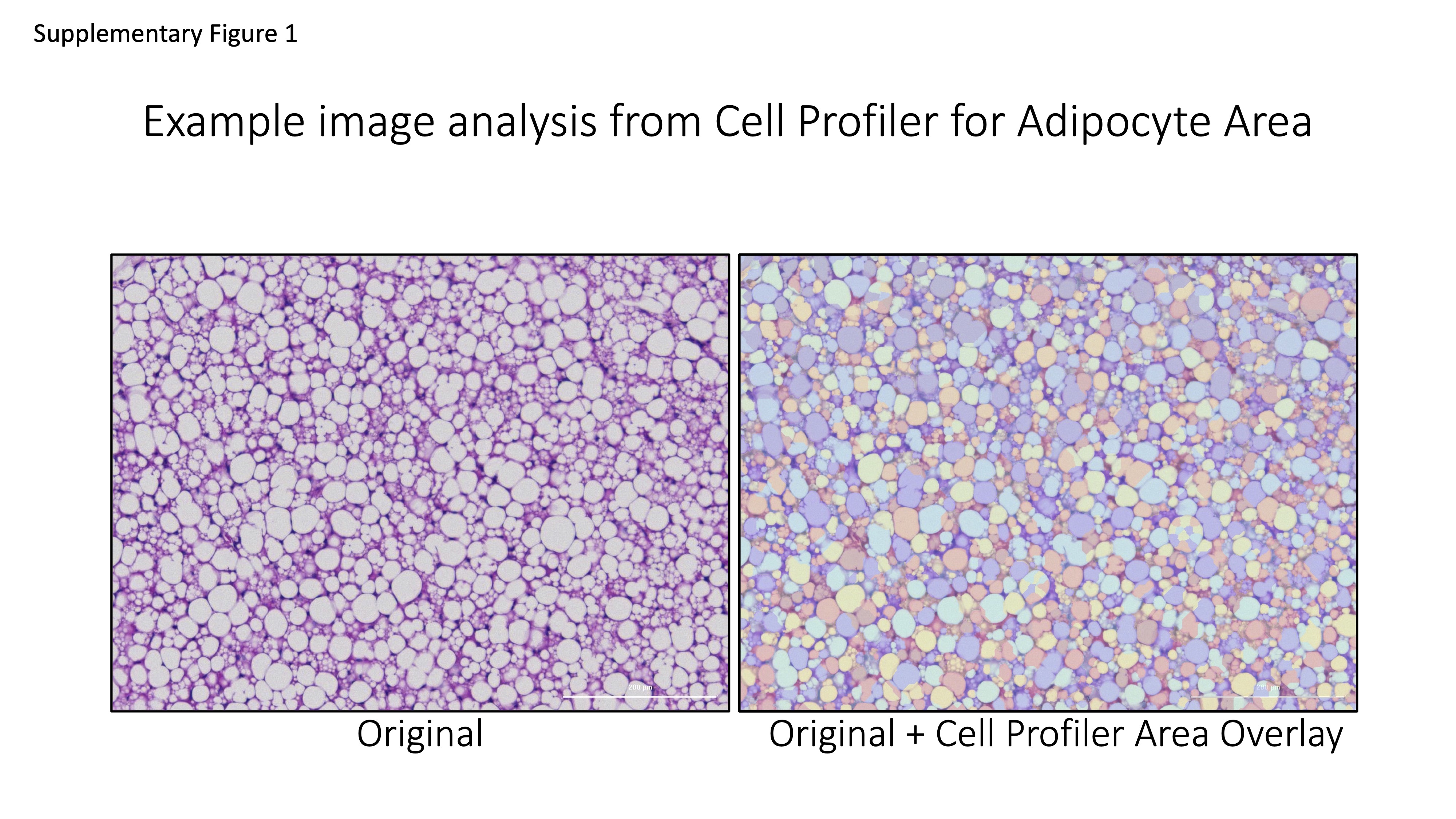

Supplement: Supplementary file 1 — Fig S1 [file PHY2-10-e15292-s002.jpg]

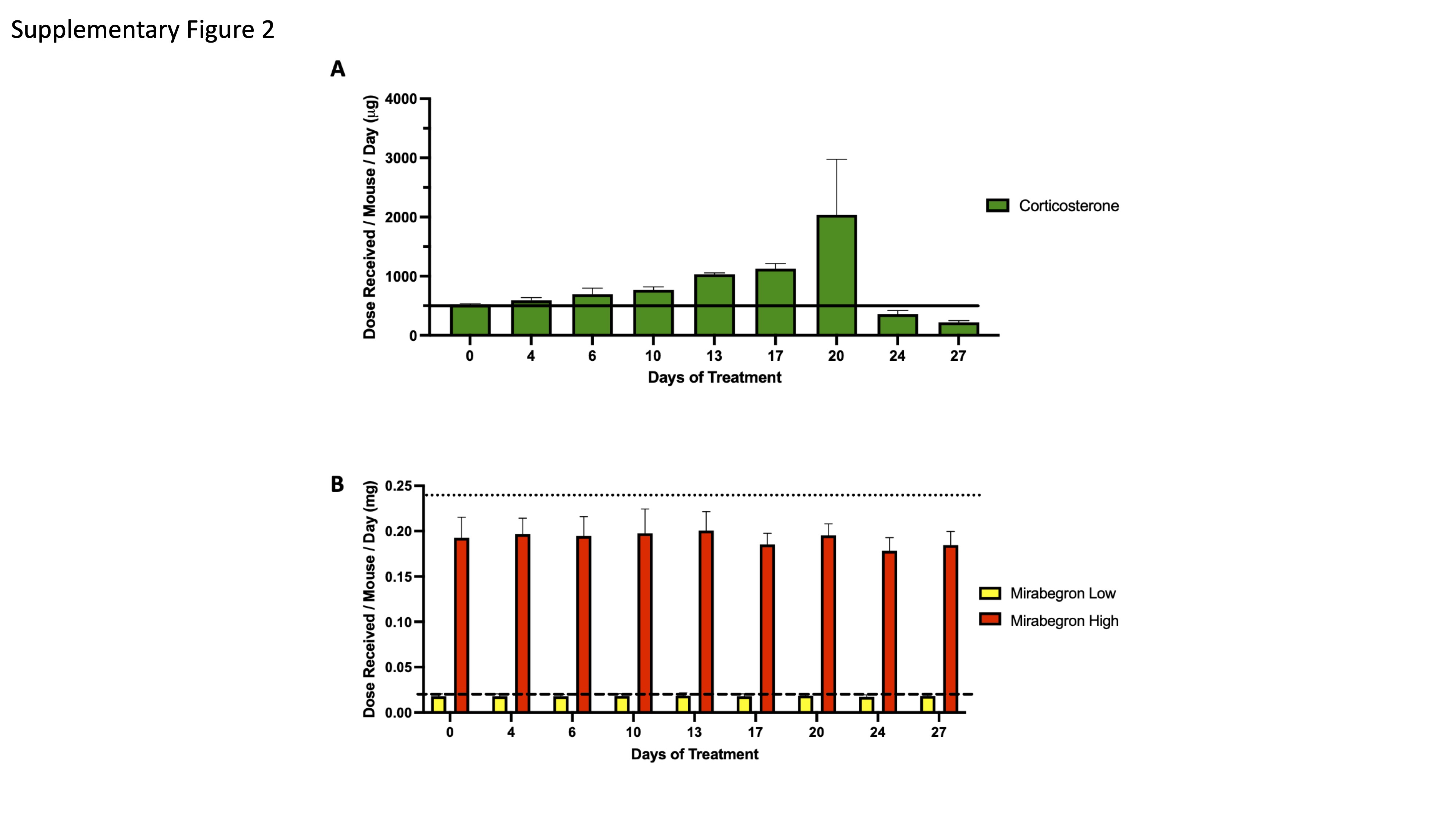

Supplement: Supplementary file 2 — Fig S2 [file PHY2-10-e15292-s004.jpg]

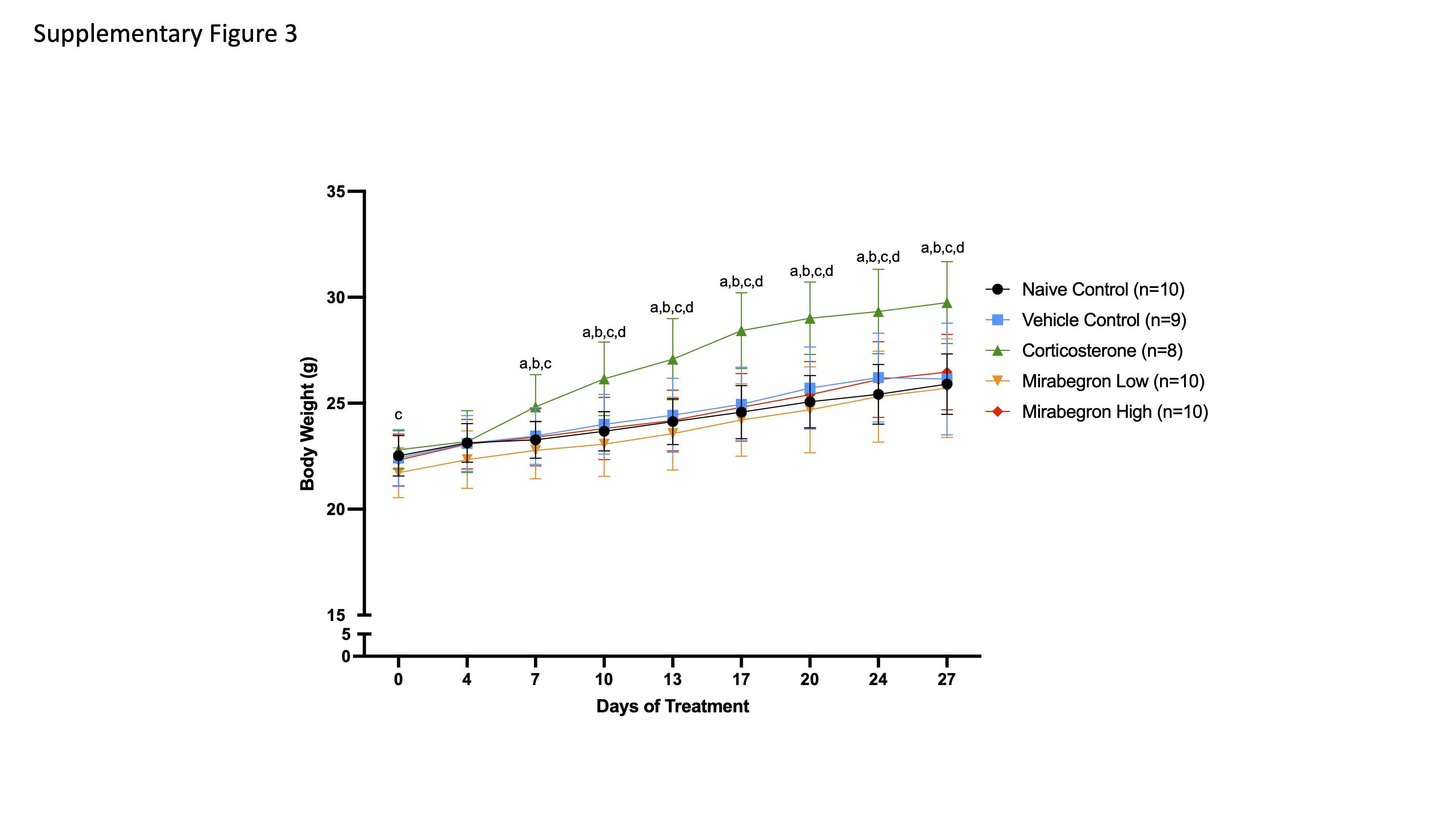

Supplement: Supplementary file 3 — Fig S3 [file PHY2-10-e15292-s001.jpg]

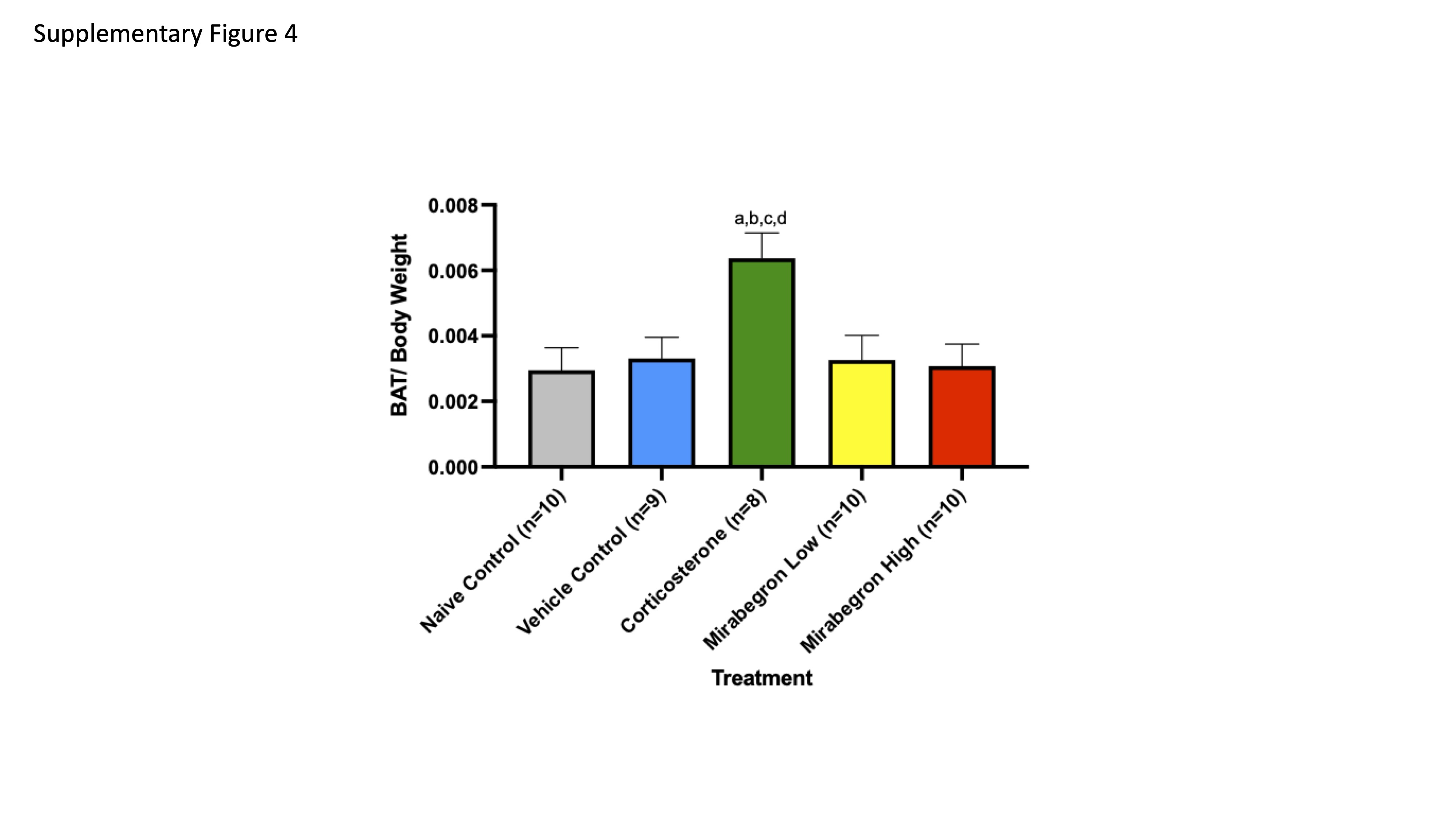

Supplement: Supplementary file 4 — Fig S4 [file PHY2-10-e15292-s003.jpg]
